# Clinical implementation of suicide risk prediction models in healthcare: a qualitative study

**DOI:** 10.1186/s12888-022-04400-5

**Published:** 2022-12-14

**Authors:** Bobbi Jo H. Yarborough, Scott P. Stumbo, Jennifer Schneider, Julie E. Richards, Stephanie A. Hooker, Rebecca Rossom

**Affiliations:** 1 Kaiser Permanente Center for Health Research, 3800 N Interstate Ave Portland, 97227 Portland, OR USA; 2 Kaiser Permanente Washington Health Research Institute, WA Seattle, USA; 3 Health Services Department, University of Washington, WA Seattle, USA; 4 HealthPartners Institute, Minneapolis, MN USA

**Keywords:** Implementation, Clinician, Artificial intelligence, Qualitative methods, Suicide risk

## Abstract

**Background:**

Suicide risk prediction models derived from electronic health records (EHR) are a novel innovation in suicide prevention but there is little evidence to guide their implementation.

**Methods:**

In this qualitative study, 30 clinicians and 10 health care administrators were interviewed from one health system anticipating implementation of an automated EHR-derived suicide risk prediction model and two health systems piloting different implementation approaches. Site-tailored interview guides focused on respondents’ expectations for and experiences with suicide risk prediction models in clinical practice, and suggestions for improving implementation. Interview prompts and content analysis were guided by Consolidated Framework for Implementation Research (CFIR) constructs.

**Results:**

Administrators and clinicians found use of the suicide risk prediction model and the two implementation approaches acceptable. Clinicians desired opportunities for early buy-in, implementation decision-making, and feedback. They wanted to better understand how this manner of risk identification enhanced existing suicide prevention efforts. They also wanted additional training to understand how the model determined risk, particularly after patients they expected to see identified by the model were not flagged at-risk and patients they did not expect to see identified were. Clinicians were concerned about having enough suicide prevention resources for potentially increased demand and about their personal liability; they wanted clear procedures for situations when they could not reach patients or when patients remained at-risk over a sustained period. Suggestions for making risk model workflows more efficient and less burdensome included consolidating suicide risk information in a dedicated module in the EHR and populating risk assessment scores and text in clinical notes.

**Conclusion:**

Health systems considering suicide risk model implementation should engage clinicians early in the process to ensure they understand how risk models estimate risk and add value to existing workflows, clarify clinician role expectations, and summarize risk information in a convenient place in the EHR to support high-quality patient care.

**Supplementary Information:**

The online version contains supplementary material available at 10.1186/s12888-022-04400-5.

## Background

Automated suicide risk prediction models derived from machine learning of electronic health records (EHR) are a relatively new innovation in suicide prevention and have potential to improve identification of individuals at high-risk of suicide attempt. Performance of these models generally ranges between 60 and 80% classification accuracy, [1–11] a measurable improvement compared to traditional clinical assessment [12]. A study in the Veterans Health Administration found that a clinical program using a validated suicide risk prediction model to identify the top 0.1% suicide risk tier was associated with more outpatient encounters, increased documentation of new suicide prevention safety plans, and fewer inpatient mental health admissions, emergency department visits, and documented suicide attempts [13]. Under the pressure of rising national suicide rates, [14] and amid forecasting of a surge in mental health service use subsequent to the COVID-19 pandemic, health care systems are interested in implementing suicide risk identification models to identify and prioritize their highest risk patients for risk assessment. Nascent research suggests that patients support this use of their EHR data but have reservations about how these models will be implemented [15].

While prediction models hold promise for suicide prevention, as of yet, there is no evidence that these models confer additional benefits beyond the routine suicide risk screening that occurs in many health systems, nor that they identify patients not otherwise identified. Further, there are practical and logistical concerns about how suicide risk models should be integrated into clinical workflows, what liabilities and responsibilities they introduce for health systems and clinicians, and how they might influence patient visits, risk communication, decision making, the clinician-patient relationship, and clinical interventions. There is almost no literature describing suicide risk model implementation in health systems [16] to guide risk model adopters and implementation planners in how to manage these pragmatic concerns.

The present study explores these concerns and describes clinicians’ and health care administrators’ expectations for and experiences with implementing a suicide risk prediction model across three health systems. The goal of this applied qualitative research was to equip potential suicide risk prediction model adopters to deploy these models in a manner that is patient-centered (a companion manuscript describes patient perspectives) [15], supports clinicians, and is sustainable.

## Methods

At one site considering future implementation of a suicide risk model, qualitative interviews focused on perceived benefits and risks associated with suicide risk prediction models. Two other sites conducted small implementation pilots, affording the opportunity to study stakeholder perspectives on the advantages and disadvantages of two different implementation approaches. Often research focuses on pre-implementation factors or observes and measures ongoing implementation. This unique design—including pre-implementation and implementation settings—allows observation of whether anticipated barriers and facilitators in the pre-implementation site are borne out in the implementation settings. Tailored interview guides based on the Consolidated Framework for Implementation Research (CFIR) [17] were used to elicit respondents’ expectations for and experiences with the suicide risk model in clinical practice, and suggestions for improving suicide risk model implementation. The Kaiser Permanente Northwest (KPNW) Research Subject Protection Office (Institutional Review Board) reviewed and approved all study materials; all participants provided informed consent and all methods were performed in accordance with the relevant guidelines and regulations.

### Settings

The settings were three healthcare systems that provide medical and specialty mental health care and insurance coverage to enrolled patient/member populations: KPNW and Kaiser Permanente Washington (KPWA) regions, each serving approximately 600,000-700,000 members in Oregon and Washington, and HealthPartners (HP) which provides care to 1.2 million patients and insurance coverage to 1.8 million members, primarily in Minnesota and western Wisconsin. In each of these settings, patients/members are primarily covered by commercial insurance or Medicare or Medicaid and are demographically similar to the service area population. All three sites are part of the Mental Health Research Network which developed the suicide risk model that was implemented in the two implementation pilots. The model predicts risk of suicide attempt within 90 days following an outpatient visit in primary care (in which a mental health diagnosis was made) or a mental health specialty clinic [11]. The model was developed and validated in seven US Health systems; in a separate study, the model was validated in an 8th health system serving American Indian/Alaskan Native patients. The eight health systems in which the model has been tested vary significantly in suicide risk screening and how suicide prevention care is organized and prioritized.

### Pre-implementation context

#### KPNW

KPNW did not implement the suicide risk prediction model and thus served as a pre-implementation site. KPNW has been implementing components of the Zero Suicide framework [18] since 2016; is participating, along with KPWA and four other large health systems, in an evaluation of the Zero Suicide model [19]; and participates in a national suicide prevention collaborative [20]. There is leadership support and a robust infrastructure at KPNW to support suicide prevention including system-wide depression and suicide risk screening using standardized instruments (Patient Health Questionnaire [PHQ-9] [21], Columbia Suicide Severity Rating Scale [C-SSRS] [22]), established depression care management and suicide prevention care pathways, outreach to high-risk patients who have missed appointments or transitioned from emergency or inpatient suicide-related care, and evidence-based suicide prevention interventions. Implementation of a suicide risk prediction model is a next step in the broader KPNW suicide prevention program.

#### KPWA

Like KPNW, KPWA participates in a national suicide prevention collaborative, has a rich local context for suicide prevention guided by the Zero Suicide framework, a well-established process for risk assessment and follow-up workflows, and a long history of use of the C-SSRS.

#### HP

At the time of their implementation pilot, HP was participating in a statewide collaboration to implement elements of the Zero Suicide model. Part of that work involved training to implement the C-SSRS in emergency department and inpatient settings. Prior to the pilot, use of the C-SSRS in the health system was not yet routine; behavioral case managers were not administering structured suicide risk assessments to patients on their caseload and had no experience with the C-SSRS.

### Implementation context at HP—an outreach-based approach

**Fig. 1:**
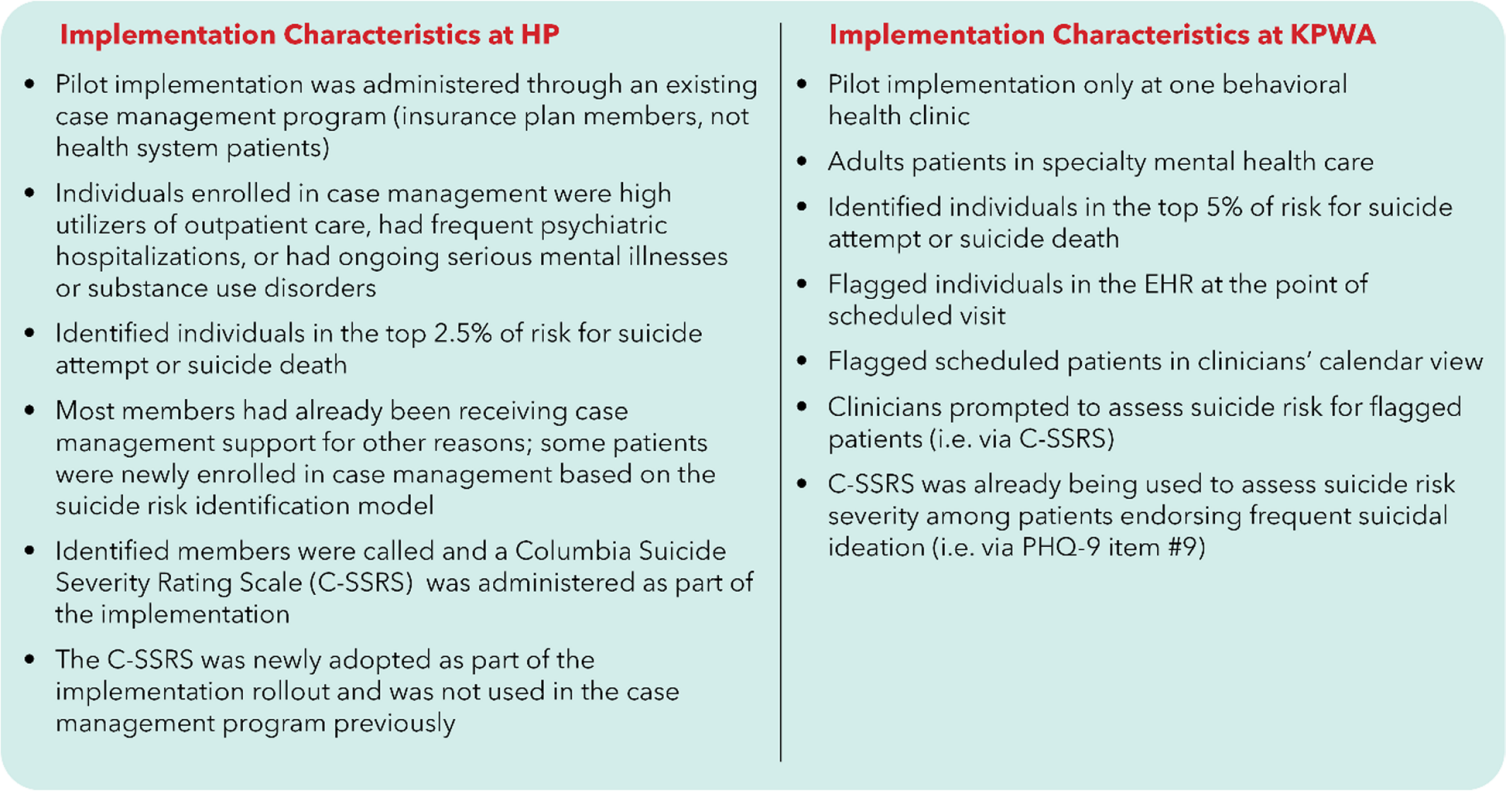
Description of implementation at HP (outreach approach) and KPWA (visit-based approach)

At HP, leadership decided to integrate the suicide risk model into an existing behavioral health case management program that served insurance plan members who were high utilizers of outpatient care, had frequent psychiatric hospitalizations, or had ongoing serious mental illnesses or substance use disorders (Fig. 1). In addition to supporting members who had opted into case management, the rationale for the outreach approach was to offer support to at-risk members who had not yet engaged with case management.

Clinical and information technology leaders created a weekly report of members on the case management patient registry who were in the top 2.5% of suicide risk and embedded the C-SSRS into the case management module in the EHR, making it easy to access and use. Multiple C-SSRS trainings by clinician experts were offered throughout implementation. Case managers also received training on crisis management, how to implement the suicide risk model within case management workflows, expectations for outreach, and how to have risk conversations and document them. Refresher trainings provided an opportunity for case managers to give feedback and for leaders to adjust the approach.

Two major implementation changes resulted from HP case manager feedback. Initially individuals identified by the suicide risk model who were not already engaged in the voluntary case management program were added to the caseloads. When case managers had difficulty connecting by phone with these members leaders limited the outreach program to members who had been active in case management in the previous two years. This improved the metric for C-SSRS completions but reduced the reach of the program. A second adjustment was responsive to case managers’ concerns that members who remained on the weekly list would be bothered by successive outreach that required C-SSRS completion. Leaders allowed case managers discretion in determining whether to conduct a C-SSRS when reaching out to members who had recently completed a risk assessment but remained on reports.

Case managers at HP were expected to outreach to any members identified on a weekly list. They did not explicitly describe the suicide risk model to members; in rare cases when a new patient questioned why they were contacted they were told HP had a new tool to help them identify members who may need additional supports.

### Implementation context at KPWA—a visit-based approach

Like at HP, implementation at KPWA included early partnership with information technology. KPWA leaders chose a visit-based implementation approach (i.e., to identify risk only among those scheduled for an upcoming visit) in a single behavioral health clinic (Fig. 1). A risk flag indicator was embedded in the EHR, in the clinicians’ schedule, to alert clinicians to scheduled patients who exceeded the 95th percentile for risk of a suicide attempt in the 90 days following the visit. The C-SSRS was later embedded as an electronic form when COVID-19 drove health systems to deliver virtual care. Engagement of clinicians involved didactic training in team huddles and distribution of a “huddle card” that summarized the rationale for implementing the suicide risk model, its empirical evidence, where the flag would appear in the EHR, clinician action being requested, sample scripting for patient communication, and answers to frequently asked questions (e.g. what data are used to calculate risk, how risk scores are updated). The strategy of training in team huddles followed by distribution of huddle cards is common practice at KPWA for deployment of innovations or practice changes.

Similar to the HP workflow, KPWA clinicians did not inform patients that they had received an alert prompting them to conduct a suicide risk assessment. At KPWA it is not unusual for patients to be asked about suicide ideation in mental health visits. Risk communication with patients was framed as “checking in” and workflows associated with the risk flag were identical to those if the risk had been identified by any means (i.e., screener, patient self-report).

### Sample

Potential interview participants included health system or behavioral health administrators with authority to implement suicide prevention initiatives and clinicians who responded to an email recruitment solicitation (KPNW) and/or participated in one of the two implementation pilots (HP, KPWA).

### Recruitment

Using purposeful sampling [23], we aimed to interview all relevant administrative stakeholders involved in implementation and up to 10 clinicians per site. We anticipated this would be a sufficient sample size to understand implementation barriers and facilitators [24] but planned to continue interviews until saturation of themes was reached. At KPNW, the two suicide prevention administrative leads were recruited directly by email. Mental health clinicians recruited by staff email newsletter volunteered to participate; a few were recommended through snowball sampling to represent specific roles in the health system (e.g., emergency psychiatric services). Due to policy, administrators and clinicians were not compensated. At HP, the three administrators most involved in implementation and the ten case managers with the most experience with the suicide risk model were invited via email; all agreed to participate, none received renumeration. At KPWA, two administrators with roles in the implementation pilot and three broader health system leaders were invited to participate. All clinicians in the clinic where the implementation pilot occurred were recruited by email. An opt-out phone number was provided; those who agreed to participate received $50 compensation.

### Interviews

Approximately 30-minute interviews were conducted between May and November 2020 by participants’ work phone and audio-recorded using HIPAA-compliant software. Three distinct but overlapping interview guides were customized to each setting. At KPNW, questions were hypothetical in anticipation of future suicide risk model implementation and focused on acceptability of the use of suicide risk prediction models and expectations or preferences. Interviews at HP and KPWA elicited administrators’ and clinicians’ experiences using a suicide risk model and inquired about their experiences and their perceptions of their patients’ experiences of risk conversations. Interview questions were guided by CFIR [17], a framework for understanding implementation context. We chose CFIR as our goal was to outline implementation facilitators and barriers based on this novel approach to identifying patients at risk for suicide and to provide initial evidence for how to move such implementation work forward. See supplemental materials for sample interview guide and Table 1 for sample interview domains. Interviewers had broad expertise in qualitative methods and were primarily female, with master’s or doctoral training in psychology (JER, SAH), public health (JS), or sociology (SPS). Interviewers were unknown to participants, interviewer motivation for the research was not disclosed; interviewers were interested in suicide prevention, health system quality improvement, and implementation research.

**Table 1:** Administrator and clinician sample interview domains

	CFIR Domains			
	Characteristics of Individuals	Inner Setting	Process	Intervention Characteristics
**Administrator interviews**				
Familiarity with risk prediction models	x			
Perceived value of risk models	x	x		
Vision and implementation plans (e.g., in which populations the model would be applied and why)			x	
Leadership support		x		
Plans for follow up of high-risk patients			x	
Communication, training plans			x	
Actual or anticipated impact on existing workflows, burden				x
**Clinician interviews**				
Understanding of risk information presented by risk model	x			
Familiarity with how risk model produce risk estimates	x			
Expectation to see individual predictors for at-risk patients				x
Whether not seeing predictors for at-risk patients is problematic				x
Expectations for using risk model over time				x
Preferences for implementation process and supports				x
Adequacy of suicide prevention resources		x		
Burden of suicide risk identification and follow up			x	x
Alert fatigue				x
Ethical considerations of suicide risk prediction models				x

### Analysis

We employed a constant comparative analysis approach [25–28] led by two trained qualitative methodologists (JS, SPS), and facilitated by Atlas.ti qualitative analysis software [29]. First, we reviewed six transcripts (two from each site) to develop a preliminary codebook. We applied the resulting codes to those six transcripts and an additional four transcripts, meeting frequently to discuss the coding progress, identify changes to the codebook (e.g., addition of new codes, clarifying definitions), and resolve coding discrepancies. All changes were retroactively applied to previously coded transcripts. Codes reflected interview questions (e.g., initial reaction to tool), content naturally emerging from the interview (e.g., patient-provider relationship), and relevant CFIR domains (e.g., intervention characteristics).

Following coding of the remaining transcripts, query reports for each code were created, read, and summarized by one of the two coding authors (JS, SPS). When coding was nearly complete, coders met with interviewers from all sites to debrief. We used CFIR domains to organize our analysis so as to be able to speak directly to potential implementation concerns, some of which were naturally brought up by clinicians in the course of our interviews while others were planned probes in the interview guide. We conducted a formal debrief interview using CFIR as our guide to assess interviewers’ understanding of clinician and administrator perspectives. This process sensitized the coders to be aware of certain topics from the different sites and served as a way of triangulating salient themes [25, 30]. Next, the full analytic team (BJY, JS, SPS) met weekly to discuss important themes and subthemes from each summarized query report, and how the results across all queries fit together as a whole. Results were derived from assessing the relative importance that clinicians and administrators attributed to critical aspects of suicide risk modeling generally and the implementation procedures more specifically. We focused on particularly salient concerns raised by clinicians across the three sites. Additionally, at the two implementing sites, we focused on opportunities for quality improvement and issues that directly impacted the work of clinicians and/or how they felt their patients would react to the use of risk models. Thematic results were shared with interviewers from each site as a means of “member checking” interpretations [25–27].

## Results

Administrators and clinicians (*n* = 13 HP, *n* = 15 KPWA, *n* = 12 KPNW) completed interviews; Table 2 displays the demographic characteristics of the sample. At the two implementation sites we were able to interview nearly everyone (HP) or everyone (KPWA) involved in implementation, feedback from the third site was largely consistent with the others, helping us to be confident we had reached saturation both in terms of sample and themes.

**Table 2:** Demographic characteristics of interview sample (self-reported)^a^

	*n* = 40
Mean Age	43 years (SD = 13)
Gender	
Female	31 (78%)
Male	8 (20%)
Transgender	1 (3%)
Race	
Asian American	3 (8%)
Native American	1 (3%)
African American	2 (5%)
White	31 (78%)
Multiple Race	3 (8%)
Ethnicity	
Hispanic/Latinx	2 (5%)
Non-Hispanic/Latinx	38 (95%)

^a^Percents may not total to 100% due to rounding

### Common implementation concerns across settings

Interviewees generally supported use of suicide risk prediction models. Most (73%) felt health systems had a responsibility to monitor suicide risk using EHR and insurance claims data; many felt they had a personal responsibility, based on their job, to monitor patients’ suicide risk. There was broad agreement that risk models should be implemented by experienced clinicians in mental health departments. More than half (60%) of interviewees had a favorable first impression of the risk model, about 26% had mixed feelings, and four participants felt the model was not helpful or had little added value. A case manager with mixed feelings reported that their assumptions of how patients might respond did not play out as expected:

*“I have had really good conversations with members where maybe in the past I didn’t directly ask about their suicidal ideation. Once I did, sometimes their answers surprised me. I was worried that members might be uncomfortable with me asking so blatantly about suicidal ideation, even though it’s super-important. However, I’ve found that most people were okay with it, and I didn’t receive the response I thought I would.” (HP)*

Those who were ambivalent or unsupportive of suicide risk models cited concerns about patient perceptions (e.g., overreach, privacy) and the lack of data to support risk model implementation.

*“I haven’t seen anything really tangible in terms of risks or benefits from a data perspective. And I haven’t heard even anecdotes of like, oh, we did outreach to this patient and, you know, we saved their life. I don’t feel connected enough to real concrete information to give more than a theoretical response… My belief is that the rewards outweigh the risks. I think it’s the right thing to do.” (KPWA)*

#### Theme: Clinicians want to be involved

Across settings, clinicians wanted to be included in discussions and planning not just told to implement something new. Clinicians who had been using the suicide risk model for a while requested refresher trainings that allowed them to give feedback; they wanted to debrief on how use of the risk model was going and to have the opportunity to request process changes that were needed. There was a perception that more involvement would increase acceptance and support of the risk model. It was unclear to many clinicians how suicide risk models fit into the numerous, ongoing suicide prevention efforts taking place in their health systems. Clinicians felt under pressure, balancing many competing demands. There was a perception that health systems were constantly rolling out new programs, while clinicians were unsure why something new was needed or what was lacking. Clinicians expressed alert fatigue and change fatigue.

*“I did have the feeling that we have lots of different workflows being assigned. Like okay, this person was identified with this algorithm. And this person was identified with this algorithm. And it just started to feel like, ‘how many different algorithms are we running right now?’ Each time a new process would be added, it just contributed to this feeling like, ‘okay, I’m going to do my best’…” (HP)*

*“The fact that we get asked to implement so many things is challenging. I mean, we are getting updates quickly on how to even do video visits and the state laws. And [clinic] things are getting changed often around this and coding…we have increasing documentation needs and documentation requirements which then often feel more like requirements for someone besides me and the patient. (KPWA)*

*“There’s an enormous amount of practice and workflow change that’s in process at the moment. And just speaking for myself and the close colleagues that I work with, any time soon any other changes are just not going to be welcome, just because the pace of everybody’s wanting to change the process all at once is just over-burdening.” (KPNW)*

#### Theme: Clinicians want more training

Some clinicians described discomfort with assessing suicide risk which created an additional emotional burden; experienced clinicians had concerns that not all clinicians were adequately trained for suicide prevention work. Some had concerns about lack of ability or confidence to conduct an in-depth risk assessment; others were concerned clinicians needed more training to avoid misunderstanding risk results or overreaction that would stigmatize patients. Commonly clinicians requested additional guidance on how to communicate with patients, including talking points.

At HP, case managers had good recall of the content of the training they received; many felt reasonably supported and well-trained when the HP pilot began. However, some who were less experienced in suicide risk assessment felt the training was insufficient, described resistance to the added workload, thought the work was better suited for mental health or primary care, and would have liked more refresher trainings. At KPWA, a huddle card was used to convey information about the risk models in lieu of additional training, which was insufficient for some clinicians. There seemed to be a discrepancy between what KPWA leaders felt they provided in terms of training and what implementing clinicians experienced. Some had a vague recall of a training, mostly focused on how to access the electronic C-SSRS; they were generally aware of the model from weekly team huddles, but felt they needed more guidance and resources. Some clinicians did not recall a training. The KPWA risk flag required a manual process to make it visible in the EHR. This was a barrier for several clinicians who needed more specific instructions for how to turn it on. As a result, some clinicians who were interviewed had not used the risk flag because they had never enabled it.

Clinicians wanted to understand the suicide risk model better. Specifically, they wanted to understand why patients were identified because they did not have access to the predictors that contributed to the risk scores. They understood that not all risk factors are available in EHR data; they were less familiar with why someone may be at risk who does not appear to be.

*“I have to say there have been a few times too, I’m like why is this person coming up?...I can’t even figure out sometimes like where that came from. If it’s diagnoses or what.” (HP)*

Some HP case managers were not surprised to see some of their clients flagged at-risk, but also felt the same patients were already getting adequate services and support so the case managers questioned the value added by the risk model.

*“[I haven’t] identified any like big barriers to using it. Other than maybe some frustration. In that it’s identifying these same people who are being identified by these other algorithms. And that are already getting support for what they need… And sometimes I think people are being identified, that the algorithm is catching them, because they are having like med changes, or additional providers. But that for them is like an indicator of positive change for them.” (HP)*

Some felt they would appreciate the risk model more if it could limit to people not already on their radar. Some KPWA clinicians also described surprise that certain clients were unexpectedly identified while others they expected were not. Mismatch between clinicians’ experiences with patients and risk model results created tension, especially because the underlying risk predictors were opaque to clinicians.

*“Yeah, so the people who have the flag I’m like, ‘okay, that makes sense they have the flag’. But I guess my bigger concern or question would be some of the people who should have flags don’t have flags… they have a ton of previous hospitalizations or attempts and don’t have a flag. … When I see who is flagged I start to get a little confused of whatever the algorithm is for selecting the people who are getting the flag. So, I have a lot of questions about it in general - I have mixed feelings about it so far.” (KPWA)*

One leader acknowledged the need to train clinicians to understand that someone could be at risk even if the risk was not apparent to the clinician.

*“It’s something that I’ve thought about a lot in terms of providers not knowing the relative risk. One piece of feedback we often get [from clinicians] when talking about the Columbia is ‘Do I have to do a safety plan when the score is three and really they’re not having suicidal ideation? It was something that happened a long time ago. They’ve had some suicidal behavior, but that’s not why they’re here today.’ According to the Columbia those folks are still at moderate risk. And our providers pushback and say, ‘I can’t do [safety planning] when that’s not why they’re here.’ But those patients really are at risk. And that’s challenging for us to talk to our providers and convince them.” (KPNW)*

#### Theme: Clinicians want liability protection

Clinicians were concerned that they could be sued for not doing something in response to the risk model, or for not acting fast enough. They perceived their access to risk information from the prediction model differently than their access to risk information from notes in the EHR or screeners. They wanted to know where their personal liability as a clinician ended.

*“Just knowing that I could be the last one to be asking about this and making sure that I’m doing everything. Like, a lot of it comes down to my clinical judgment and trusting what the patient says and that’s what I have to go off of. So yeah, there is a lot of liability, especially for very high risk patients that I make the right clinical decision and have it documented of why I made the decision I did.” (KPWA)*

*“I would say if I’m the only one accessing it that’s a major liability. If I’m accessing it with an actual care team then I would say the liability would be less or at least it would be on all of us to make sure that we’re following up.” (KPWA)*

*“And then another thing, I think would be like what is the liability? If you did know that somebody was at risk and you didn’t respond or didn’t do anything, what would be the liability of this? …Maybe I was sick for a few days. Something happened. And it shows that they’re at risk and nothing was done, and that person completes a suicide, well, I’m liable. [Organization] is liable.? … And so then what does that mean in terms of liability?” (KPNW)*

#### Theme: Clinicians want clear expectations

Clinicians want clear instructions for what to do in cases where patients are unavailable or unwilling to engage in risk discussions or when patients have chronic ideation or remain high risk because of unchangeable risk factors. There was ambivalence about whether repeatedly screening such patients was helpful or harmful and a desire to minimize burden on clinicians as well as patients who may become fatigued repeatedly answering standardized risk assessment questionnaires.

*“It was frustrating, especially in the beginning. You would get a [C-SSRS] and then the next month, you’d get the same person. And the next month, you’d get the same person. And I’m like, for some people, they’re just going to keep being on the list. And that was frustrating at the beginning because I do not need to make extra phone call attempts for somebody who I already talked about this twice a month and who already has all these people involved. Somewhere along the line [the health system] just put a note saying just FYI or use your discretion. And that was really good because it kind of took the pressure off of feeling like I need to make calls that didn’t really have a lot of purpose or meaning.” (HP)*

#### Theme: Clinicians want suicide risk information consolidated within the EHR

Finally, to make risk assessments more efficient and less burdensome to themselves and for patients, clinicians recommended that everything related to suicide assessment—all historic suicide risk screening scores, history of attempts, family history, and other relevant information—should appear in a consolidated place in the EHR. Clinicians cited the trauma for patients having to repeatedly re-explain their family or personal suicide history to new clinicians and the time involved in finding past risk scores and reading chart notes to ascertain risk history as justification for investment in a suicide risk module.

### Limitations of each implementation approach

#### Outreach as implemented at HP

An obvious limitation of the outreach approach is that it is less effective when clinicians are unable to reach members on their lists. This was cited as the biggest barrier by all but one of the HP interviewees.

*“Every month, I get maybe half of my outreach. And then the other half, I don’t…For me it’s only been getting ahold of the member. I can’t even think of another barrier, like I haven’t had a problem with getting the questions answered. I haven’t had any complaints about that.” (HP)*

Case managers were also concerned when they lacked a prior relationship with members on their at-risk list, which made rapport building more challenging during outreach calls. KPNW clinicians acknowledged that assessing and interpreting risk would be more difficult without a shared understanding of the patient’s history to put the risk in context.

The HP approach revealed the importance of timing. The at-risk list was generated weekly which meant that members reappeared weekly. This elicited a fairly strong negative reaction by the case managers who sometimes felt that nothing significant had changed since their previous outreach. They worried about overburdening members, some of whom were identified while still receiving inpatient care or were well-established in outpatient care and already receiving all available resources. As described above, the initial outreach goals and protocol were adjusted in response.

#### Visit-based approach as implemented at KPWA

One limitation of the visit-based approach as described by a few KPWA clinicians was difficulty balancing priorities in time-limited visits. KPWA clinicians had to negotiate time to address acute patient concerns while allowing enough time to conduct the required risk assessment. KPWA clinicians reported that upon noticing a risk alert, the C-SSRS was accessible and easy to use but still required ample time to complete and document it, connect people to care, and organize resources. Recommendations were made to prompt clinicians to document the C-SSRS; these suggestions were echoed by KPNW clinicians.

**Fig. 2:**
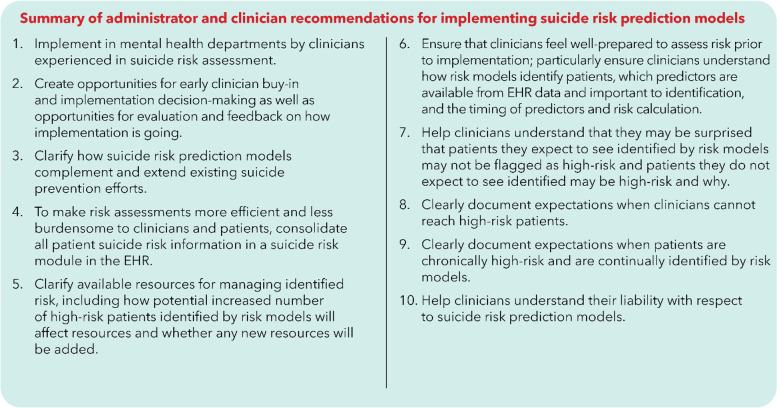
Administrator and clinician recommendations for implementation of suicide risk prediction models

Administrator and clinician recommendations for implementing suicide risk prediction models are summarized in Fig. 2.

## Discussion

Generally, case managers and clinicians were supportive of using suicide risk models because they felt identification of high-risk patients were important public health and health system priorities. Consistent with previous research [15], they felt risk models should be implemented by well-trained clinicians in mental health settings. There was ample leadership support for suicide risk model implementation and the inner settings for both implementation pilots were enriched by multiple suicide prevention efforts, but these typical facilitators were experienced as barriers by some clinicians who described alert and change fatigue associated with constantly changing demands. Some clinicians were less convinced of the relative advantage of the risk model compared to existing suicide prevention initiatives. Others felt most identified patients already had all available resources and supports and there was little left to offer them. Enthusiasm was further dampened by clinicians’ inability to understand individuals’ risk predictors and some concern when clinician expertise and experience with particular patients was at odds with risk model results. Previously published survey research has highlighted clinician preferences for knowing which risk factors contribute to an individual’s predicted suicide risk as well as the lower clinician likelihood to respond to risk model outputs when they are incongruous with clinicians’ preconceived risk assessments [31].

Each of the observed implementation approaches had distinct advantages and disadvantages. A health systems’ suicide prevention goals—reaching high-risk patients including those who may have disengaged from care or identifying the highest risk among those engaged in services—should drive implementation decision making and choice of an outreach-based, visit-based, or hybrid model. Perhaps the greatest benefit of an outreach approach is the possibility of reaching individuals who might not otherwise present for care, but the experience at HP showed that these patients may be difficult to reach. Adopters need to consider how to manage unreachable patients and need to help clinicians understand the limits of their personal liability when patients cannot be reached.

In contrast, a behavioral health visit-based approach only impacts the patient population receiving mental health care, potentially missing many at-risk patients. However, an advantage of a visit-based approach is that, unless the patient is new to the clinician, clinicians will have an established relationship, cumulative knowledge of the patient’s risk and protective factors, and an understanding of their personal context and risk trajectory over time which may be particularly important for people with chronic suicide ideation or past attempts. Another advantage of the visit-based implementation pilot was the seamless workflow that mimicked existing workflows. It was imperceptible to patients.

In both implementation pilots, patients were unaware that clinicians received a prompt to ask them about their suicide risk. There were no explicit discussions about the use of a suicide risk prediction model. There was agreement among clinicians that knowing who was at risk helped them to prioritize the risk discussion, but none felt it necessary to mention the model. Clinicians preferred autonomy to adjust the conversation to the unique patient/call/visit needs. It is likely that this organic approach and communication style mitigated any alarm that patients may have experienced should a more direct approach have been used.

Two very important implementation process considerations became clear, particularly in the HP outreach pilot. First, adopters must consider the appropriate frequency to generate risk scores and second, they must clearly operationalize how to follow up with persistently at-risk patients who have immutable risk factors and have recently completed risk assessments. In determining frequency of generating risk scores, health care systems should explicitly consider how frequently risk model inputs are refreshed. Even with frequent updates, individual level risk factors may change infrequently (e.g., suicide attempts) or not at all (e.g., gender, historical diagnoses). When risk inputs remain relatively stable, individuals may be consistently identified even when their risk has not changed since the last assessment. Decisions must be made about how frequently to follow up with stably high-risk patients. Careful consideration of these implementation decisions is important to avoid overburdening clinicians and patients with frequent suicide risk assessments which may not be warranted.

Several areas were identified to improve suicide risk model implementation. These included recommendations to integrate the suicide risk prediction model and associated workflows within the broader framework for suicide prevention and helping clinicians to understand their value relative to other health system efforts. Health systems adopting a suicide risk model should also plan additional pre-implementation clinician training focused on how clinicians will know whether their patient has been identified as high risk and, if that information is conveyed in the EHR, clear directions for finding it; what a high risk score means, including how it was derived, how clinicians are expected to communicate risk to patients, including talking points; and for less experienced clinicians, how to conduct a thorough risk assessment. Health systems should also give clinicians the opportunity to give feedback throughout implementation. Finally, clinicians requested the integration of risk assessment tools into the EHR so that a risk score, suicide screening scores, safety plans and related interventions are all accessible from a common, efficient location.

This project has several strengths. We were able to observe two distinct implementation approaches that resulted organically from health system decisions. Observations began early in the implementation process, allowing for early and mid-stream observations. The pre-implementation sites allowed us to compare administrator and clinician expectations and preferences to what was actually occurring in the two implementation settings. This unique design allowed cross-validation of findings and robust support for clinician preferences. Importantly, it also allowed us to see whether certain concerns raised in the pre-implementation site were borne out in the implementation settings. To support credibility, rigor, and trustworthiness of our interview findings, interviews were conducted by trained qualitative staff; utilized interview guides shaped by CFIR domains to establish consistency and thoroughness in data collection; persisted until saturation was reached (at the two implementing sites we interviewed nearly all clinicians involved in the pilot studies); and were analyzed with a team-based, constant comparative approach. Using the CFIR domains in analysis allowed us a framework to organize our findings to focus on actionable, quality improvement opportunities and decisions adopters would need to consider. For example, focusing on the construct of relative advantage (the advantage of implementing in one way versus an alternative way) facilitated recognition of site implementation differences. This allowed us to compare the advantages, disadvantages, and reasons for various implementation decisions, for example, the decision to take a visit-based or outreach approach. Nevertheless, a couple limitations should be noted. In all the settings, suicide prevention was acknowledged as a health system priority and the Zero Suicide framework had been implemented to varying degrees. Other settings without this enriched suicide prevention context may experience unique barriers, not identified here, related to the implementation of suicide risk models. Not all relevant stakeholders may have been interviewed, possibly limiting the diversity of experiences and opinions. Additionally, patient preferences and opinions are not included. We interviewed 62 patients for this project and their preferences are being analyzed separately for a future publication.

## Conclusion

Suicide risk identification tools may be an important component of suicide prevention efforts; they are rapidly advancing toward implementation. Guidance on how to implement such programs has been scant. Adopters should consider overall health system suicide prevention goals and available resources; engage clinicians in the implementation process early if possible; provide adequate training including how the risk model is expected to add value to existing suicide prevention initiatives, how it is meant to augment not replace clinical decision making, and why certain patients may or may not be flagged. It is important to clarify clinicians’ role expectations and liability, support clinicians with summarized risk information in the EHR, and provide the opportunity for clinicians to give and receive feedback on the process of risk model implementation.

## Supplementary Information

**Additional file 1.**

## Data Availability

The datasets generated or analyzed during the current study are not publicly available because asking participants to consent to interview transcripts or a publicly shared dataset derived from interview content could compromise the quality of the research by resulting in selection on unobserved heterogeneity—the requirement for data sharing could influence the types of individuals willing to participate in the study or the type of information participants are willing to share. Further, maintaining our study participants’ confidentiality is of paramount importance. It may not be possible to sufficiently anonymize or redact transcripts to prevent the possibility of deductive disclosure. Our qualitative interview guides are freely available to any interested researchers. We will also provide full code books upon request. Requests for interview guides or code books should be sent to Dr. Yarborough.
