# Supplementary material for: Clinical implementation of suicide risk prediction models in healthcare: a qualitative study
Source: BMC Psychiatry. 2022 Dec 14;22:789. doi: 10.1186/s12888-022-04400-5 (PMC9748385; doi:10.1186/s12888-022-04400-5)
Supplement: Supplementary file 1 — Additional file 1. [file 12888_2022_4400_MOESM1_ESM.pdf]

CLINICAL IMPLEMENTATION OF A SUICIDE RISK ALGORITHM

Interview Guide for Health Care Clinicians

Introduction

Thank you for agreeing and taking time to meet today. We're going to be talking about risk prediction in the context of health care. You may be aware that there is an increasing interest in the idea of using data to predict which patients are at risk for adverse health outcomes, for primary or secondary prevention. Algorithms derived from machine learning are increasingly being used across health care in areas such as cancer or cardiovascular disease prevention, to predict health risks and drive clinical care; but how these tools should be implemented in everyday clinical practice at the point of patient care or during preventive outreach and the human factors that influence their use, remain understudied. We'd like to talk with you about risk prediction in health care and specifically about suicide risk identification models and how they might be implemented in your health system/practice.

General familiarity with and use of risk identification tools

Does your department currently use any type of risk prediction or identification tool?

> If yes, can you describe what it is and how you use it? (*Probe: make sure they specifically describe how they interact with any tool currently in use*)

How well do you think patients understand the concept of future risk?

> How might this understanding, or lack of understanding, affect patient care?

Vision for suicide risk identification tool

I'd like to tell you a little bit about an algorithm that we've developed to identify suicide risk. Using electronic health records data, including patient characteristics, diagnoses, prescriptions, and use of services/treatments, we are able to identify patients at the highest risk of making a suicide attempt (or dying by suicide) in the 3 months following an outpatient health care visit.

Tell me your thoughts about the possibility of using a suicide risk identification algorithm, based on electronic health records data, with your patients/clients.

> How could you see using this type of information on suicide risk to improve health care?

If an algorithm told you that your patient was in the top 2% of risk, what would that mean to you? What do you think you would need to do with that information?

> If I told you that although your patient was in the top 2% of risk (i.e., they were in a high-risk group) their risk of actually dying by suicide was only 6%, what would you make of that information? Would that surprise you? Does that change what you would think you needed to do with the risk information? If so, in what way?

If [site] were to use these algorithms to identify at-risk patients, how would you like to be made aware of a patient's elevated suicide risk?

> *Only ask about prompts below when participant brainstorming is exhausted, and then ask about advantages/disadvantages of each approach:*
>
> I'm going to tell you about three possible approaches that are either under consideration or already being implemented in other health systems; I'd like to hear your thoughts about each of them.

Automated best practice alerts that fires during a patient visit. What do you think of this approach? At what point either before or during the visit would you need that information? Do you have any concerns about alert fatigue, if so, what are they?

A risk calculator initiated by you, the clinician. What do you think of this approach? Should it be external or internal to Epic? Do you have any concerns about putting the burden of identification on the clinician (that is, rather than having a program run and populate the EHR automatically)?

Panel support tool monitored by a care manager, independent of patient visits. What do you think of this approach? Do you have any concerns about someone other than you contacting your patients about suicide risk, if so, what are they? How do you anticipate your patients would react (either positively or negatively) to having someone other than you outreach to them about elevated suicide risk?

Besides knowing that a patient is at high risk for suicide, what other information, if any, would you need in order to communicate effectively with the patient?

What if the factors that contributed to a patient's risk were not available to you? That is, if the algorithm could not tell you specifically what contributed to the patient's risk but only that they were at-risk? Would that be a problem?

What if the algorithm identified the patient as being in the top 5th percentile for suicide risk in the next 3 months? How would you communicate that information to the patient? Or would you?

Perceived value of and thoughts on implementing a suicide risk identification algorithm

I'd like to hear your thoughts about any potential barriers to implementing this type of intervention at [site]. By barriers, we mean anything that you anticipate might get in the way of launching, implementing or sustaining suicide risk identification algorithms.

Interviewer: Make sure to probe for barriers at the intervention, provider, organization, or system level.

Next, I'd like to hear your thoughts about any potential facilitators to implementing this type of intervention at [site]. By facilitators, we mean anything that might make it easier to launch, implement, or maintain the algorithms.

Interviewer: Make sure to probe for facilitators at the intervention, provider, organization, or system level.

What, if any, suicide prevention strategies are currently in place in [site] in your department? How might an intervention based on a suicide risk identification algorithm compare to these existing programs (advantages, disadvantages)?

Interviewer: Make sure to probe for what they are currently doing, how they feel it is going, and how they think the intervention components compare to that.

[If not already mentioned] Now I would like to ask you about characteristics of the providers and staff in your department that might affect launching this intervention, implementing it from day to day, and maintaining it over time. Tell me about staff factors which may affect implementation of this algorithm.

[If not already mentioned] What about characteristics of your organization that might affect how suicide risk models are implemented?

What resources or support would be needed to effectively implement in your setting?

What about characteristics of your larger health care context (i.e., local, state, or national-level) that might affect implementing? Tell me about factors pertaining to your larger system context which may affect implementation.

Interviewer: Make sure to probe for policies, regulations, laws that may be important considerations.

Anything else you'd like to add or that I haven't asked you about regarding using a suicide risk identification algorithm in your clinical practice?

<u>***Final demographic questions***</u>

We just have a few last questions that we are required to provide to our research funder.

1. Which of the following best describes you? (include all that apply)
    a. White
    b. Black or African American
    c. Native Hawaiian or Other Pacific Islander
    d. Asian
    e. American Indian/Alaska Native
    f. Other (please specify) _____________
2. Which of the following best describes your ethnicity?
    a. Hispanic or Latino
    b. Not Hispanic or Latino
3. What is your gender
    a. Male
    b. Female
    c. Other (please specify) ____________
4. What is your age (in years)? _____

Thank you.
